# Associations between Life’s Essential 8 and post-stroke depression and all-cause mortality among US adults

**DOI:** 10.1186/s40001-024-01834-3

**Published:** 2024-04-12

**Authors:** Ruicong Ma, Junting Song, Yanchun Ding

**Affiliations:** 1 Department of Cardiology, The Second Hospital of Dalian Medical University, No.467 Zhongshan Road, Shahekou District, Dalian, 116021 Liaoning People’s Republic of China; 2 Department of Neurology, The Second Hospital of Dalian Medical University, Dalian, 116021 Liaoning People’s Republic of China

**Keywords:** PSD, Life's Essential 8, NHANES, All-cause mortality

## Abstract

**Background:**

Depression is the common mental disease after stroke. Our objective was to investigate the correlation of Life’s Essential 8 (LE8), the recently updated evaluation of cardiovascular health, with the occurrence of post-stroke depression (PSD) and all-cause mortality among United States (US) adults.

**Methods:**

Participants with stroke were chosen from the National Health and Nutrition Examination Survey (NHANES) between 2005 and 2018. The relationship between LE8 and the risk of PSD was assessed through weighted multiple logistic models. A restricted cubic spline was employed for the examination of correlations. To demonstrate the stability of the results, sensitivity analysis and subgroup analysis were carried out. Furthermore, Cox regression models were used for the correlation between LE8 and all-cause mortality.

**Results:**

In this study, a total of 1071 participants were included for analysis. It was observed that LE8 score and PSD risk shared an inverse relationship in per 10 points increase [OR = 0.62 (0.52–0.74, *P* < 0.001)] in logistic regression models. The analysis of restricted cubic spline demonstrated approximately a noticeable inverse linear association between LE8 score and PSD risk. Sensitivity analysis verified the stability of the findings. Moreover, no statistically significant interactions were identified in subgroup analysis. A reverse association between LE8 score and all-cause mortality was also observed with a 10-point increase [HR = 0.85 (0.78–0.94, *P* < 0.001)] in cox regression models.

**Conclusions:**

A negative correlation was discovered between LE8 score and PSD and all-cause mortality risk among US adults. We need to conduct large-scale prospective studies to further validate our results.

## Introduction

Stroke is a severe cerebrovascular disease, imposing significant economic burden and presenting substantial challenges globally, notably in the United States [1]. In 2020, stroke claimed the lives of approximately 6.6 million individuals, making it the second most common cause of death [2]. In addition, stroke ranked as the third leading cause of disability, further highlighting its significant impact on individuals and societies. What is particularly concerning is the rising incidence of stroke among younger age groups, including both the young and middle-aged populations [3]. Post-stroke depression (PSD) is a common neuropsychological disorder in individuals who have suffered from stroke. This condition is characterized by emotional deterioration and noticeable decline in interest [4]. The incidence of PSD is high in patients (18–33%) [5, 6]. PSD can also affect mental health and has the potential to hinder an individual's ability to engage in rehabilitation. Furthermore, it can diminish medication adherence and increase the risk of drug abuse, which may increase the risk of disability and death [7, 8]. In spite of effective efforts in PSD prevention and treatment, managing PSD remains challenging [9]. Consequently, reducing the incidence of PSD holds immense importance in the field of public health.

Cardiovascular health (CVH) is closely related to psychological health. Numerous scientific studies validate the connection among the brain, mind, heart, and body, which have the potential to have either positive or negative effect on cardiovascular health [10]. Psychological health may affect CVH through various direct or indirect mechanisms, such as increased inflammation, disrupted glucose, lipid metabolism and impaired autonomic nervous system function [11]. Moreover, mental health can affect lifestyle habits, which in turn can have an impact on CVH [12]. In addition, the impact of CVH on PSD cannot also be ignored. Stroke patients are often accompanied by a variety of cardiovascular risk factors, including smoke, obesity, dyslipidemia, etc. [13]. These factors were involved in the occurrence of PSD. Furthermore, certain unhealthy lifestyle habits, including smoking, inadequate dietary choices and inactive behavior, can also contribute to the increased risk of PSD [14–16].

The American Heart Association (AHA) introduced Life’s Essential 8 (LE8) as a method to measure CVH recently. CVH is composed of two parts, including health behaviors (diet, physical activity, nicotine exposure and sleep health) and health factors [body mass index (BMI), non-high-density-lipoprotein (HDL) cholesterol, blood glucose and blood pressure] [17]. Some studies have found that LE8 is associated with the reduced risk of various diseases, such as chronic kidney disease, abdominal aortic calcification, dementia and nonalcoholic fatty liver disease [18–21].

The association between LE8 and PSD risk is still not well-understood, despite its close relationship with many diseases. Therefore, it is important to investigate this relationship to improve the prevention and treatment of PSD.

## Materials and methods

### Data source and study participates

**Fig. 1:**
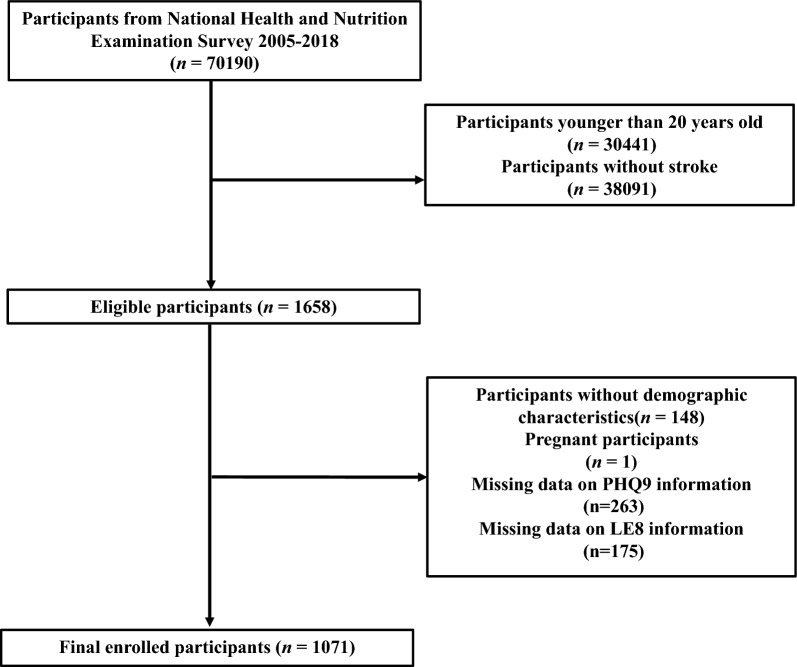
The flow chart of participant selection

We carried out this study by utilizing data from the National Health and Nutrition Examination Survey (NHANES) database available at www.cdc.gov/nchs/nhanes.com. The purpose was to evaluate the health conditions of individuals aged 20 and older in the United States. Data samples were collected from different states and counties within the United States. These samples were obtained from all NHANES participants from 2005 to 2018 (*n* = 70,190), we excluded participants whom younger than 20 years (*n* = 30,441), those without stroke or missing stroke information (*n* = 38,091), those without demographic characteristics (*n* = 148), pregnant participants (*n* = 1) and participants missing data on 9-item Patient Health Questionnaire (PHQ-9) information (*n* = 263) and LE8 information (*n* = 175). According to previous NHANES research, the inquiry regarding stroke was as follows: “Has a doctor or other health professional ever told you that you had a stroke?”. Individuals were classified as stroke participants if they responded affirmatively to this query. The analysis sample comprised 1071 participants in total. The screening process details were illustrated in Fig. 1.

### Measurement of LE8

The American Heart Association (AHA) introduced Life’s Essential 8 (LE8) as a method to measure CVH recently. CVH is composed of two parts, including health behaviors (diet, physical activity, nicotine exposure and sleep health) and health factors (BMI, non-HDL cholesterol, blood glucose and blood pressure). The total LE8 score was derived by calculating the average of the ratings for each of the 8 indicators, which were scored on a scale ranging from 0 to 100 points. According to the American Heart Association (AHA), individuals with LE8 score of 80 or greater were identified as high cardiovascular health (CVH). Those with LE8 score ranging from 50 to 79 were considered as moderate CVH, while individuals with LE8 score below 50 were categorized as low CVH [17].

### Assessment of depression symptom

The PHQ9 scale was used to evaluate depression symptom. A cutoff value of 10 or above on the PHQ-9 scale was recognized as depression [22].

### Demographic information

Demographic information was obtained from the NHANES database, which contained data on age (in years), sex (categorized as male or female), racial/ethnic background (including white, black, Mexican and others), educational attainment (categorized as less than high school, high school, and post-high school education), marital status (married, never married and divorced) and poverty income ratio (≤ 1.30, 1.31–3.49 and ≥ 3.50).

### Diagnosis of comorbidities

Coronary heart disease, congestive heart failure and chronic obstructive pulmonary disease (COPD) were diagnosed based on medical history. To test for hepatitis B surface antigen, healthcare professionals may use the VITROS HBsAg test, the VITROS HBsAg kit on the VITROS ECi/ECiQ Immunodiagnostic System and VITROS 3600 Immunodiagnostic System, and the VITROS Immunodiagnostic Product HBsAg Calibrator. As for hepatitis C ribonucleic acid (RNA), the COBAS Amplicon HCV Monitor test is used. The COBAS Amplicon HCV Monitor version 2 0 (v2.0) is an in vitro nucleic acid amplification test for the quantification of HCV RNA in human serum or plasma on the COBAS Amplicon analyzer. If these test indicators are positive, they indicate a hepatitis virus infection [23]. We applied diabetes evaluation criteria: doctor diagnosis as diabetes, HbA1c ≥ 6.5%, fasting glucose ≥ 7.0 mmol/L, random blood glucose ≥ 11.1 mmol/L, 2 h OGTT blood glucose ≥ 11.1 mmol/L, or being treated with diabetes drugs and insulin [24].

### Mortality

To assess the mortality, we paired the National Death Index data with the mortality information for the period ending on December 31, 2019.

### Statistical analysis

First, we divided the data into two groups: non-PSD and PSD. Means corresponding standard error of the mean (mean ± SEM) were used to express continuous variables, whereas proportions with their respective 95% CI were employed for categorical variables. Frequency is used to represent comorbidities. To ascertain variations between the two groups, independent sample *t* tests were conducted for continuous variables, while Chi-square tests were utilized for categorical variables. Statistical significance was considered at *P* values < 0.05. Next, we calculated each component’s mean (95% CI) and compared the differences between non-PSD and PSD. Third, we conducted weighted logistic regression analyses to examine the correlation between LE8 and PSD. We constructed two models: Model I and Model II. Model I was unadjusted model. Model II was adjusted for age, sex, race, education levels, marital, poverty income ratio, diabetes, coronary heart disease, congestive heart failure, COPD and viral hepatitis. Results were presented as odds ratios (ORs) with 95% CIs. To examine the correlation between LE8 and PSD, we employed a restricted cubic spline method. Various investigators have employed distinct analytic approaches, including weighted analysis methods and unweighted methods. While NHANES utilizes intricate sampling methods to improve the representativeness and applicability of findings, there are instances, where the deductions derived from weighted and unweighted analyses may deviate. Consequently, we performed a sensitivity analysis using unweighted regression analysis to revalidate our findings in this investigation. In addition, subgroup examination was employed to further ensure the consistency of results. Finally, cox regression analyses were used to investigate the correlation between LE8 and all-cause mortality.

## Results

### The baseline characteristics of participants

A total of 1071 screened participants were involved, of which 202 were diagnosed with PSD. The baseline characteristics of all participants, including age, sex, race, education levels, marital, poverty income ratio and LE8 score are presented in Table 1. Table 1 shows significant differences in clinical characteristics between the PSD group and non-PSD group. Compared with the non-PSD group, patients with PSD showed higher PHQ9 score, lower levels of education and poverty income ratio. Moreover, the proportion of congestive heart failure and COPD are higher in the PSD group. To enhance comprehension regarding the relationship between LE8 and the risk of PSD, we also conducted a comparison of the LE8 score components between groups with PSD and without PSD (Table 1). In the PSD group, the LE8 score was found to be significantly lower compared to the non-PSD group. It is noteworthy that in the LE8 score components, the non-PSD group had significantly higher HEI‐2015 diet score, sleep health score, nicotine exposure score, physical activity score, body mass index score and blood lipids score than the PSD group (*P* < 0.05).

**Table 1:** Clinical characteristics of study population

Variables	Overall (*n* = 1071)	Non-PSD (*n* = 869)	PSD (*n* = 202)	*P* value
Age, %	64.33 ± 0.60	65.67 ± 0.66	58.41 ± 1.02	< 0.001***
Sex, %				
Female	56.13 (49.59,62.67)	54.97 (50.70,59.24)	61.23 (52.42,70.04)	0.21
Male	43.87 (38.64,49.11)	45.03 (40.76,49.30)	38.77 (29.96,47.58)	
Race/ethnicity, %				
White	73.06 (64.21,81.91)	73.50 (69.48,77.53)	71.10 (62.81,79.39)	0.74
Black	13.65 (11.44,15.85)	13.16 (10.85,15.47)	15.81 (10.58,21.05)	
Mexican	4.17 (3.01, 5.34)	4.10 (2.83,5.36)	4.51 (1.94,7.09)	
Others	9.12 (6.70,11.53)	9.24 (6.74,11.74)	8.58 (3.77,13.38)	
Education levels, %				
Less than high school	22.22 (18.88,25.55)	21.95 (18.70,25.20)	23.41 (17.42,29.39)	0.02*
High school or equivalent	32.22 (27.24,37.20)	30.21 (26.31,34.10)	41.15 (32.41,49.88)	
College or above	45.56 (39.91,51.22)	47.85 (43.42,52.28)	35.45 (26.26,44.63)	
Marital status, %				
Married	59.98 (53.06,66.89)	60.61 (56.10,65.11)	57.19 (48.40,65.98)	0.43
Never married	5.90 (4.22, 7.58)	5.40 (3.57, 7.24)	8.11 (4.33,11.88)	
Divorced	34.12 (29.30,38.95)	33.99 (29.53,38.46)	34.70 (26.36,43.04)	
Poverty income ratio, %				
≤ 1.30	30.09 (26.17,34.02)	27.59 (23.78,31.40)	41.18(31.14,51.22)	0.003**
1.31–3.49	45.59 (39.11,52.08)	45.55(41.08,50.02)	45.79(37.10,54.48)	
≥ 3.50	24.31 (20.06,28.56)	26.86(22.52,31.20)	13.03(6.48,19.57)	
PHQ-9 score	5.19 ± 0.22	2.90 ± 0.12	15.32 ± 0.32	< 0.001***
LE8 score	57.00 ± 0.63	58.89 ± 0.67	48.63 ± 1.25	< 0.001***
HEI-2015 diet score	37.53 ± 1.15	39.30 ± 1.32	29.70 ± 2.08	< 0.001***
Sleep health score	75.42 ± 1.17	79.02 ± 1.18	59.49 ± 3.04	< 0.001***
Nicotine exposure score	65.15 ± 1.44	69.00 ± 1.61	48.09 ± 2.98	< 0.001***
Physical activity score	48.01 ± 2.05	49.90 ± 2.08	39.64 ± 4.72	0.03*
Body mass index score	52.04 ± 1.49	54.12 ± 1.48	42.93 ± 3.12	< 0.001***
Blood glucose score	68.90 ± 1.32	69.31 ± 1.46	67.06 ± 2.79	0.47
Blood pressure score	48.77 ± 1.31	48.59 ± 1.44	49.56 ± 3.02	0.77
Blood lipids score	61.41 ± 1.18	62.92 ± 1.25	54.62 ± 3.63	0.04*
DM, %	463 (43.23)	375 (43.15)	88 (43.56)	0.63
Coronary heart disease, %	192 (17.93)	146 (16.80)	46 (22.77)	0.11
Congestive heart failure, %	189 (17.65)	137 (15.77)	52 (25.74)	0.004
COPD, %	134 (12.51)	94 (10.82)	40 (19.80)	0.01
Viral hepatitis, %	38 (3.55)	30 (3.45)	8 (3.96)	0.86

Continuous data were presented as the mean ± SEM, category data were presented as the proportion and 95% confidence interval. Frequency is used to represent comorbidities

*SEM* Standard Error of the Mean, *PSD* post-stroke depression, *PHQ-9* 9-item Patient Health Questionnaire, *HEI* Healthy Eating Index, *LE8* Life’s Essential 8, *DM* diabetes, *COPD* chronic obstructive pulmonary disease

^***^P value < 0.001, **P value < 0.01, *P value < 0.05

### Relationship between LE8 score and PSD

In the unadjusted logistic regression analysis (Table 2), we observed a negative correlation between LE8 score and PSD, represented as a continuous variable, with an odds ratio (OR) (per 10 points increase) of 0.60 (95%CI 0.52–0.70). In Model II, LE8 score was significantly negatively correlated with PSD (per 10 points increase) (OR = 0.62, 95%CI 0.52–0.74). Compared with the high CVH, the higher risk of PSD was showed in the moderate CVH (OR = 8.08, 95%CI 1.46–19.94) and low CVH (OR = 19.72, 95%CI 3.69–34.62) in Model II.

**Table 2:** Weighted logistic regression analysis on the association between LE8 score and PSD

	Model I		Model II	
	OR [95% CI]	*P* value	OR [95% CI]	*P* value
Continuous LE8 score (per 10 points increase)	0.60 (0.52,0.70)	< 0.001***	0.62 (0.52,0.74)	< 0.001***
High CVH (80–100)	Reference	–	Reference	–
Moderate CVH (50–79)	7.84 (1.95,21.50)	0.004**	8.08 (1.46,19.94)	0.02*
Low CVH (0–49)	22.22 (5.93,43.27)	< 0.001***	19.72 (3.69,34.62)	< 0.001***

Data are presented as OR (95% CI). Model I: unadjusted model. Model II adjusted for age, sex, race, education levels, marital, poverty income ratio, DM, coronary heart disease, congestive heart failure, COPD and viral hepatitis

*LE8* Life’s Essential 8, *PSD* post-stroke depression, *CVH* cardiovascular health, *DM* diabetes, *COPD* chronic obstructive pulmonary disease

^***^P value < 0.001, **P value < 0.01, *P value < 0.05

### Restricted cubic spline

**Fig. 2:**
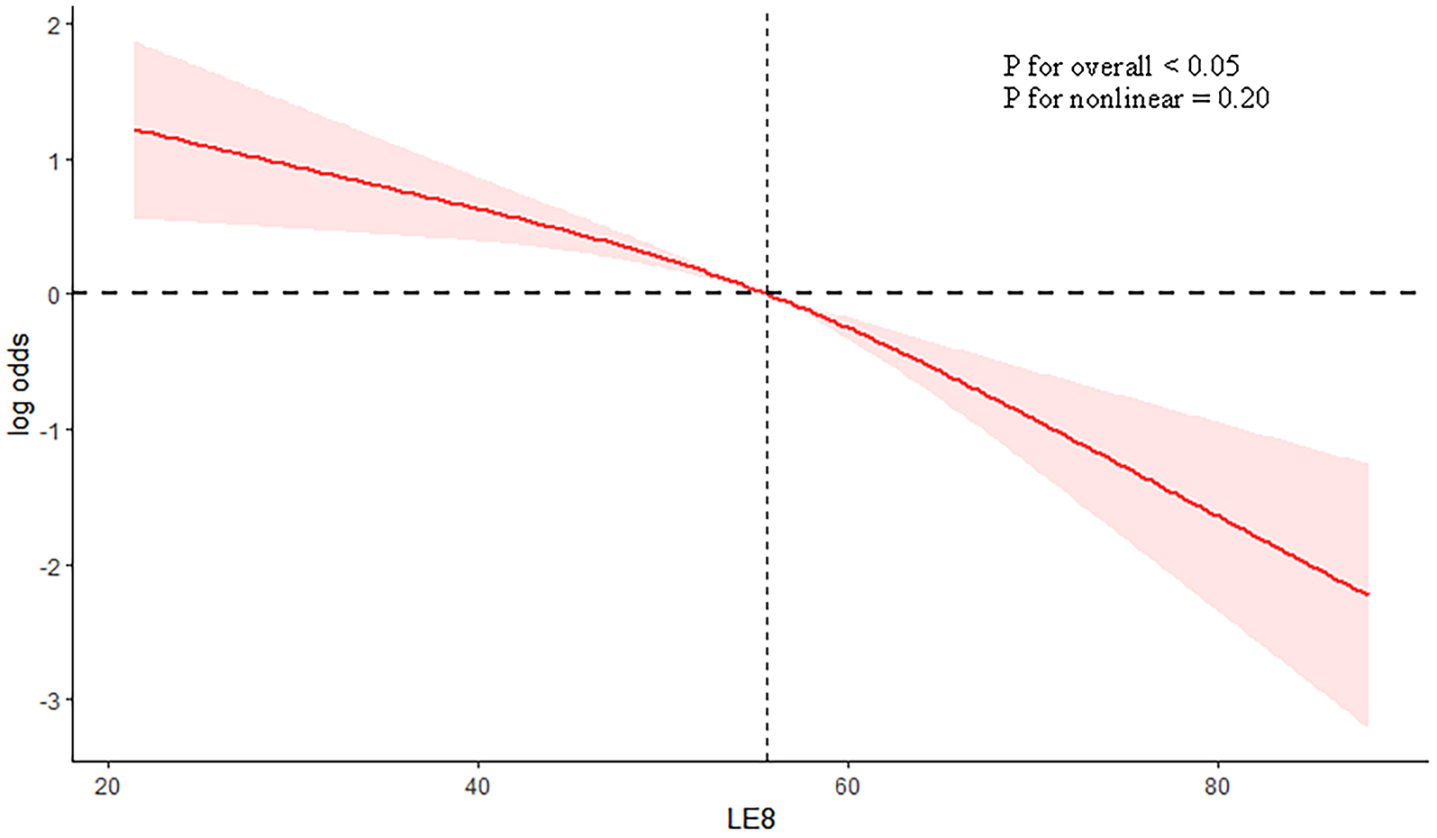
The correlation between LE8 score and the risk of PSD

**Fig. 3:**
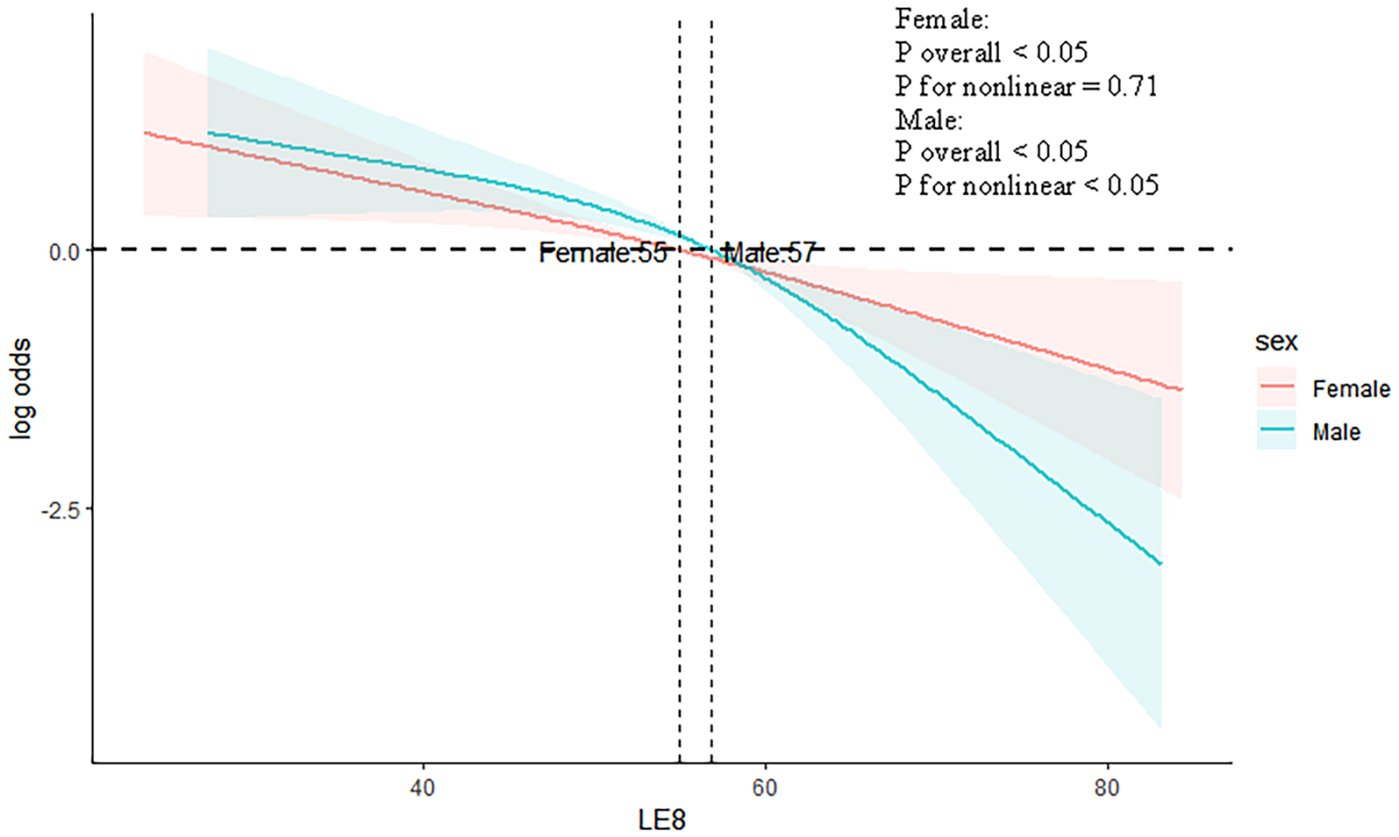
Association of LE8 score with PSD in a restricted cubic spline model stratified by sex

A restricted cubic spline was used to examine the association between LE8 score and PSD. The findings indicated a linear inverse relationship approximately between LE8 score and the risk of PSD (*P* for nonlinear = 0.20). As LE8 score rose, there was a substantial decrease in the risk of PSD (Fig. 2). In addition, the non-linear association was detected between LE8 score and depression in male individuals (*P* for non-linear < 0.05) and the linear link approximately was observed in female individuals (*P* for non-linear = 0.71) (Fig. 3).

### Sensitivity analysis

Similarly, sensitivity analysis adopting unweighted logistic analysis reveals that the higher risk of PSD was showed in the moderate CVH (OR = 4.51, 95%CI 1.23–15.62) and low CVH (OR = 8.89, 95%CI 2.37–31.02) in Model II compared with high CVH (Table 3). These results suggest a consistent inverse relationship between LE8 score and PSD.

**Table 3:** Unweighted logistic regression analysis on the association between LE8 score and PSD in sensitive analysis

	Model I		Model II	
	OR [95% CI]	*P* value	OR [95% CI]	*P* value
Continuous LE8 score (per 10 points increase)	0.66 (0.58,0.74)	< 0.001***	0.68 (0.59,0.78)	< 0.001***
High CVH (80–100)	Reference	–	Reference	–
Moderate CVH (50–79)	2.84 (1.02,11.87)	0.08	4.51 (1.23,15.62)	0.04*
Low CVH (0–49)	6.60 (2.35,27.65)	0.002**	8.89 (2.37,31.02)	0.01*

Data are presented as OR (95% CI). Model I: unadjusted model. Model II adjusted for age, sex, race, education levels, marital, poverty income ratio, DM, coronary heart disease, congestive heart failure, COPD and viral hepatitis

*LE8* Life’s Essential 8, *PSD* post-stroke depression, *CVH* cardiovascular health, *DM* diabetes, *COPD* chronic obstructive pulmonary disease

^***^P value < 0.001, **P value < 0.01, *P value < 0.05

### Subgroups analysis

We conducted subgroup analysis to examine the possible link between LE8 score and PSD among diverse subgroups categorized by age, sex, race, education level, marital and poverty income ratio (Table 4). The influence of LE8 score on PSD did not vary among the subgroups.

**Table 4:** Subgroup analysis of multi-variable adjusted association of LE8 score with the risk of PSD

Variable name	Non-PSD	PSD	*p* value	*p* for interaction
Age				
20–59 years	Reference	0.95 (0.93,0.97)	< 0.001	0.70
60 years	Reference	0.95 (0.93,0.97)	< 0.001	
Sex				
Male	Reference	0.94 (0.92,0.96)	< 0.001	0.13
Female	Reference	0.96 (0.94,0.98)	< 0.001	
Race				
White	Reference	0.94 (0.93,0.96)	< 0.001	0.11
Black	Reference	0.98 (0.95,1.00)	0.06	
Mexican American	Reference	0.97 (0.93,1.00)	0.08	
Other	Reference	0.94 (0.90,0.98)	0.01	
Education				
Less than high school	Reference	0.97 (0.94,0.99)	0.02	0.48
High school	Reference	0.95 (0.93,0.97)	< 0.001	
College or above	Reference	0.95 (0.92,0.97)	< 0.001	
Marital				
Married	Reference	0.95 (0.93,0.96)	< 0.001	0.68
Never married	Reference	0.95 (0.91,1.00)	0.04	
Divorced	Reference	0.96 (0.94,0.98)	< 0.001	
Poverty income ratio				
≤ 1.30	Reference	0.96 (0.95,0.98)	< 0.001	0.15
1.30–3.49	Reference	0.95 (0.93,0.98)	0.001	
≥ 3.50	Reference	0.92 (0.89,0.96)	< 0.001	

Each stratification was adjusted for age, sex, race, education, marital and poverty income ratio

*LE8* Life’s Essential 8, *PSD* post-stroke depression

### Relationship between LE8 score and all-cause mortality

We also used cox regression model to evaluate the association between LE8 score and all-cause mortality. We observed a negative correlation between LE8 score and all-cause mortality, represented as a continuous variable, with a hazard ratio (HR) (per 10 points increase) of 0.85 (95%CI 0.78–0.94). Compared with the high CVH, the higher risk of all-cause mortality was showed in the moderate CVH (HR = 1.46, 95%CI 0.93–2.27) and low CVH (HR = 1.80, 95%CI 1.16–2.78) in Model II (Table 5).

**Table 5:** Weighted cox regression analysis on the association between LE8 score and all-cause mortality in stroke participants

	Model I		Model II	
	HR [95% CI]	*P* value	HR [95% CI]	*P* value
Continuous LE8 (per 10 points increase)	0.92 (0.85,0.99)	0.04*	0.85 (0.78,0.94)	< 0.001***
High CVH (80–100)	Reference	–	Reference	–
Moderate CVH (50–79)	1.77 (1.00,3.13)	0.08	1.46 (0.93,2.27)	0.10
Low CVH (0–49)	1.82 (1.08,3.31)	0.042*	1.80 (1.16,2.78)	0.01*

Data are presented as HR (95% CI). Model I: unadjusted model. Model II adjusted for age, sex, race, education levels, marital, poverty income ratio, DM, coronary heart disease, congestive heart failure, COPD and viral hepatitis

*LE8* Life’s Essential 8, *CVH* cardiovascular health, *DM* diabetes, *COPD* chronic obstructive pulmonary disease

***P value < 0.001, **P value < 0.01, *P value < 0.05

## Discussion

Previous studies have found that maintaining CVH has a beneficial effect in preventing various diseases. Specifically, our findings demonstrate a negative association between LE8 score and PSD after accounting for confounding factors in the adult population of the United States. The relationship between LE8 score and PSD follows a linear pattern approximately, as depicted by the fitted smoothing curves. Interestingly, the effect of LE8 score on PSD does not differ significantly among different subgroups. In addition, we also observed a negative correlation between LE8 score and all-cause mortality.

Consistent with previous studies, better CVH can reduce the incidence of post-stroke depression. Chronic inflammation is a significant factor in the development of cardiovascular diseases. Research has shown that unhealthy eating habits often contribute to the occurrence of chronic inflammation in the body, which plays a vital role in PSD [25]. Some studied have indicated that a pro-inflammatory diet has the potential to increase oxidative stress levels and trigger immune dysregulation, thereby potentially amplifying inflammatory response [26, 27]. Unfavorable dietary patterns can result in elevated levels of inflammatory markers in the body, consequently increasing the risk of depression [28]. The consumption of pro-inflammatory diets can enhance the generation of reactive oxygen species (ROS), which results in cellular dysfunction and worsens neuroinflammation [29]. Research has shown that higher dietary quality is associated with a lower incidence of depression [30]. Similarly, this study suggests that the Healthy Diet Index score, as part of the LE8 score, is significantly elevated in non-PSD patients. A recent study on 1619 stroke patients found that poor sleep quality is a risk factor for post-stroke depression [31]. Another study also showed that the incidence of PSD is closely related to sleep duration. It is not affected by sex [32]. This study also found that the sleep health score of PSD patients was significantly worse than those of non-PSD patients. In addition, other unhealthy lifestyles are also involved in the occurrence and development of post-stroke depression, such as tobacco consumption and lack of physical activity [33–35]. In addition, recent studies have indicated that metabolic syndrome was closely related to PSD [36]. During metabolic syndrome, excessive secretion of pro-inflammatory factors by adipose tissue can result in an inflammatory response, which in turn can lead to neurological impairment [37]. The infiltration of inflammatory cells adds to the generation of reactive oxygen species, leading to oxidative stress, lipid peroxidation, protein oxidation and DNA damage within neural tissues [38, 39]. These may explain the significant correlation between LE8 score and PSD.

PSD involves multiple mechanisms, including inflammation, immune response, circadian rhythm disorders, sleep disorders, obesity, etc. Furthermore, the impact of social factors on PSD cannot be ignored. Low income and low education levels may also be involved in the occurrence of depression [40].

In this study, it was showed that LE8 score is still independently related to PSD after adjusting these social factors. In terms of economic costs and daily life, post-stroke depression brings a significant burden to patients. A healthy lifestyle is the foundation for reducing the occurrence of PSD and is also easily accepted by the public. However, previous studies have mainly focused on single factors that affect PSD, without considering comprehensive factors. LE8 score is a comprehensive and easily applicable assessment tool recently proposed by AHA, which can promote the assessment of patients' ideal health status in clinical settings and guide their rehabilitation treatment. Our research provided important reference value for the rehabilitation treatment of stroke patients. In addition, the NHANES data is designed through complex, multi-stage probability sampling to ensure the robustness of the results. In this study, the non-linear association was detected between LE8 score and depression in male individuals and the linear link approximately was observed in female individuals. These are points worth paying attention to in this study.

This research demonstrates, for the first time, the correlation between LE8 and PSD and all-cause mortality. These findings could potentially provide valuable insights for the prevention and treatment of PSD in the adult population of the United States. However, there are some limitations in this study. First, it is a cross-sectional study, meaning that it cannot establish a causal relationship between LE8 score and PSD. This highlights the need for further research to conduct longitudinal studies that can provide more conclusive evidence. Second, stroke was diagnosed according to a history of stroke which may lead to subjective bias. Third, pseudobulbar affect (PBA), is a condition commonly occurring in neurological patients and often mistakenly diagnosed as mood disorders. The duration of PBA is often short. In addition, the lack of medical history, physical examination, electromyography, Computed Tomography, Magnetic Resonance Imaging information and evaluation of a single PHQ9 scale may lead to selection bias. Finally, some studies showed that previous stroke, pain disorders, pre-existing depression or anxiety, were associated with an increased risk of PSD. Moreover, stroke severity and differences in stroke subtype or classification, i.e., ischemic stroke or intracerebral hemorrhage, TOAST classification, different brain locations of stroke, or post-stroke functional impairment are the important determinants for PSD in stroke survivors. However, we need to conduct large-scale prospective studies to further validate our results due to the lack of information in the NHANES database.

## Conclusion

A negative approximately linear correlation was discovered between LE8 score and PSD and all-cause mortality risk among adults in the United States. However, further prospective studies are still needed to reveal their relationship.

## Data Availability

The data sets generated during the current study are available in database (https://www.cdc.gov/nchs/nhanes/).

## References

[1] GBD 2019 Diseases and Injuries Collaborators. Global burden of 369 diseases and injuries in 204 countries and territories, 1990-2019: a systematic analysis for the Global Burden of Disease Study 2019. Lancet. 2020;396(10258):1204–22. 10.1016/S0140-6736(20)30925-9 PMC7567026 33069326

[2] Feigin VL, Owolabi MO (2023). Pragmatic solutions to reduce the global burden of stroke: a World Stroke Organization-Lancet Neurology Commission. Lancet Neurol.

[3] Mensah GA (2023). Global burden of cardiovascular diseases and risks, 1990–2022. J Am Coll Cardiol.

[4] Jeong S (2023). Post-stroke depression: epigenetic and epitranscriptomic modifications and their interplay with gut microbiota. Mol Psychiatr.

[5] Liu Z (2017). Malondialdehyde: a novel predictive biomarker for post-stroke depression. J Affect Disord.

[6] Hackett ML, Pickles K (2014). Part I: frequency of depression after stroke: an updated systematic review and meta-analysis of observational studies. Int J Strok.

[7] Kumar S (2017). Sobering news about post-stroke depression. Lancet Psychiatr.

[8] Kang HJ (2018). Impact of acute phase depression on functional outcomes in stroke patients over 1 year. Psychiatr Res.

[9] Owolabi MO (2023). Global synergistic actions to improve brain health for human development. Nat Rev Neurol.

[10] Levine GN (2021). Psychological health, well-being, and the mind-heart-body connection: a scientific statement from the American Heart Association. Circulation.

[11] Ladwig KH (2022). Mental health-related risk factors and interventions in patients with heart failure: a position paper endorsed by the European Association of Preventive Cardiology (EAPC). Eur J Prev Cardiol.

[12] Miller MA, Howarth NE (2023). Sleep and cardiovascular disease. Emerg Top Life Sci.

[13] Zhu J (2023). Prioritizing quality measures in acute stroke care : a cost-effectiveness analysis. Ann Intern Med.

[14] Ren W (2016). The effect of cigarette smoking on vitamin D level and depression in male patients with acute ischemic stroke. Compr Psychiatr.

[15] Su JJ (2023). Effects of mind-body exercise on physical and psychosocial well-being of stroke patients: a systematic review and network meta-analysis. Geriatr Nurs.

[16] Cherian L (2022). Western diet associated with increased post-stroke depressive symptoms. J Nutr Sci.

[17] Lloyd-Jones DM (2022). Life’s essential 8: updating and enhancing the american heart association’s construct of cardiovascular health: a presidential advisory from the American Heart Association. Circulation.

[18] Zhang Y (2023). Age-dependent interaction between Life’s Essential 8 and chronic kidney disease: a national cross-sectional analysis. Prev Med.

[19] Cai Z (2023). Associations between Life’s Essential 8 and abdominal aortic calcification among middle-aged and elderly populations. J Am Heart Assoc.

[20] Beydoun HA (2023). Cardiovascular health, infection burden, and incident dementia in the UK Biobank. Alzheimers Dement.

[21] He P (2023). A healthy lifestyle, Life’s Essential 8 scores and new-onset severe NAFLD: a prospective analysis in UK Biobank. Metabolism.

[22] Kroenke K, Spitzer RL, Williams JB (2001). The PHQ-9: validity of a brief depression severity measure. J Gen Intern Med.

[23] Chen X, Zeng Z, Xiao L (2023). The association between periodontitis and hepatitis virus infection: a cross-sectional study utilizing data from the NHANES database (2003–2018). Public Health.

[24] Ma R (2023). Association between composite dietary antioxidant index and coronary heart disease among US adults: a cross-sectional analysis. BMC Public Health.

[25] Wang YB (2023). The association between diet quality, plant-based diets, systemic inflammation, and mortality risk: findings from NHANES. Eur J Nutr.

[26] Wang X (2023). Association of dietary inflammatory potential, dietary oxidative balance score and biological aging. Clin Nutr.

[27] Vissers LE (2016). The relationship between the dietary inflammatory index and risk of total cardiovascular disease, ischemic heart disease and cerebrovascular disease: findings from an Australian population-based prospective cohort study of women. Atherosclerosis.

[28] Lai JS (2014). A systematic review and meta-analysis of dietary patterns and depression in community-dwelling adults. Am J Clin Nutr.

[29] He Q (2023). Lactoferrin alleviates Western diet-induced cognitive impairment through the microbiome–gut–brain axis. Curr Res Food Sci.

[30] Wang K (2021). Higher HEI-2015 score is associated with reduced risk of depression: result from NHANES 2005–2016. Nutrients.

[31] Fan XW (2022). Impact of persistent poor sleep quality on post-stroke anxiety and depression: a national prospective clinical registry study. Nat Sci Sleep.

[32] Liu F (2021). Impact of sleep duration on depression and anxiety after acute ischemic stroke. Front Neurol.

[33] Li S (2022). Evaluation of depression status and its influencing factors in convalescent elderly patients with first-episode stroke. Asian J Psychiatr.

[34] Lau SCL, Tabor Connor L, Baum CM (2023). Motivation, physical activity, and affect in community-dwelling stroke survivors: an ambulatory assessment approach. Ann Behav Med.

[35] Li C (2022). Treatment effect of exercise training on post-stroke depression in middle-aged and older adults: a meta-analysis. Int J Geriatr Psychiatr.

[36] Zhou X (2023). Study on insulin resistance and ischemic cerebrovascular disease: a bibliometric analysis via citespace. Front Public Health.

[37] Wang M (2020). Amino acid metabolism, lipid metabolism, and oxidative stress are associated with post-stroke depression: a metabonomics study. BMC Neurol.

[38] Wen L (2023). The predictive role of early inflammation and oxidative stress and the dynamics of cytokines networks in post-stroke depression. J Affect Disord.

[39] Nabavi SF (2014). Oxidative stress and post-stroke depression: possible therapeutic role of polyphenols?. Curr Med Chem.

[40] Sienkiewicz-Jarosz H (2010). Predictors of depressive symptoms in patients with stroke-a three-month follow-up. Neurol Neurochir Pol.

